# Termination of trastuzumab in HER2-positive metastatic breast cancer patients who received trastuzumab beyond progression

**DOI:** 10.1038/s41598-023-35715-2

**Published:** 2023-05-31

**Authors:** Izzet Dogan, Esra Aydin, Nijat Khanmammadov, Nail Paksoy, Pinar Saip, Adnan Aydiner

**Affiliations:** Department of Medical Oncology, Istanbul University Institute of Oncology, Millet Street/Fatih, Istanbul, 34093 Turkey

**Keywords:** Cancer, Cancer

## Abstract

The purpose of the study was to assess the prognosis of HER2-positive metastatic breast cancer patients who received trastuzumab beyond progression and investigate the predictors of complete response. HER2-positive metastatic breast cancer patients who received long-term trastuzumab were included in the study. Predictors of complete response were analyzed with binary regression analysis. The prognosis of patients who had their trastuzumab-based treatment terminated was assessed. Eighty patients were involved in the study. The patients were received with trastuzumab for a median of 62 months (12–191). A complete response was observed in 60 (75%) patients. The median duration to development of complete response was found as 14.8 months (2.4–55). In logistic regression analysis: using endocrine therapy with trastuzumab (p = 0.04), menopausal status (p = 0.03), and the number of metastatic sites (p = 0.01) were found to be statistically significant factors for a complete response. Trastuzumab-based therapy of fifteen patients was terminated, six (40%) patients continued to receive an aromatase inhibitor, and nine (60%) patients were followed up without treatment. After termination of trastuzumab, at a median follow-up of 32 months (11–66), recurrence was detected in two (13.3%) patients. We detected that menopausal status, the number of metastatic sites, and using endocrine therapy with trastuzumab were predictors of complete response in HER2-positive metastatic breast cancer patients who received long-term trastuzumab-based therapy. We observed that HER2-positive metastatic breast cancer patients may be completely cured with trastuzumab-based therapy. There are no defined criteria for termination of trastuzumab treatment in this selected patient group. It is necessary to confirm our data with multicenter studies involving a large number of patients.

## Introduction

Breast cancer is the most seen malignancy in females, and the treatment approach differs according to its molecular subtypes. HER2 (ERBB2), a known proto-oncogene, is located at the long arm of human chromosome 17 whose overexpression, and hence their receptors, is present in about 20–30% of breast cancers as a result of amplification of the chromosomal region 17q12-21^1^. The HER2 receptor is involved a role in cell growth, differentiation, and angiogenesis^2^. Although the HER2 receptor is a negative prognostic factor in breast cancer, it is a positive predictive factor for anticancer treatment response^3^. HER2 receptor positivity is evaluated by in situ hybridization and immunohistochemistry (IHC) methods. To date, many treatment drugs such as trastuzumab, pertuzumab lapatinib, and trastuzumab emtansine have been developed for the treatment of patients with HER2 positive breast cancer or other solid tumors^4^.

Approximately 5% of breast cancer patients are presented with metastatic disease at diagnosis time, also some of the early-stage breast cancer patients may progress to metastatic disease^5^. With trastuzumab-based therapy, disease recurrence can be reduced by 50% in patients with HER2-positive early-stage breast cancer^6^. Although trastuzumab, pertuzumab and taxane combination was used as the standard treatment in HER2-positive metastatic disease in the first series, some patients can only use trastuzumab as anti-HER2 therapy due to treatment cost or toxicity. In patients with HER2-positive metastatic cancer, cytotoxic chemotherapy drugs such as docetaxel, capecitabine, vinorelbine, and gemcitabine can be used in combination with trastuzumab^7^. In case of progression under trastuzumab-based treatment, the continuation of treatment with a different cytotoxic chemotherapy and trastuzumab combination or other anti-HER2 based treatments such as lapatinib, trastuzumab emtansine and trastuzumab deruxtecan has been shown to be effective in disease control^8–13^.

In metastatic cancer patients, complete recovery with treatment is rarely possible. In HER2-positive metastatic breast cancer patients, it has not been clearly defined how long the treatment will continue after complete response to long-term trastuzumab-based therapy. In this study, we aim to investigate the factors predicting treatment-related complete response in patients who receive trastuzumab beyond progression and to evaluate the prognosis of patients who terminated treatment after long-term complete response with trastuzumab-based therapy.

## Materials and methods

### Patients inclusion and data collection

The study was designed retrospectively. The research was carried out in accordance with good clinical practice recommendations. The patients to be included in the study were determined through the hospital data processing system. The patients included in the study consisted of patients who were diagnosed and treated in a single tertiary oncology center outpatient clinic between 2000 and 2020. Only HER2-positive metastatic breast cancer patients who received trastuzumab-based therapy beyond progression were included in the study. The pathological, clinical, and radiological features of the patients included in the study were recorded. The disease stages of the patients at the time of diagnosis were made according to the TNM classification. Estrogen receptor (ER) and progesterone receptor (PR) were evaluated by IHC in a standardized pathology laboratory. Patients with a score of 3 with HER2 receptor IHC and patients with positivity with fluorescence in situ hybridization (FISH) were also considered positive.

All surgical, chemotherapy, hormonal, and radiotherapy treatment approaches applied to the patients were recorded. The patients used trastuzumab at a loading dose of 8 mg/kg and a maintenance dose of 6 mg/kg. In addition to trastuzumab, different types of chemotherapy agents such as taxane (paclitaxel or docetaxel), platinum (cisplatin or carboplatin), gemcitabine, vinorelbine, capecitabine were given to the patients, in standardized doses. Tamoxifen and aromatase inhibitors (anastrozole, letrozole, and exemestane) were used as hormonal therapy. In addition, luteinizing hormone-releasing hormone (leuprolide or goserelin) was used in hormone-positive premenopausal patients. Radiological and clinical response evaluations were performed every 3 months in patients. Treatment responses were classified according to the RECIST 1.1 criteria. Univariate and multivariate analyzes were performed to identify factors predicting complete response. In addition, cardiac evaluation was performed with echocardiography every 3 months, and treatment-related side effects were recorded.

In case of progression under trastuzumab-based therapy, trastuzumab was continued, and the concomitant chemotherapy agent was changed. The time from initiation of trastuzumab-based therapy to complete cessation of trastuzumab was considered progression-free survival (PFS). Chemotherapy agents used together with trastuzumab were recorded. The time from onset of metastatic disease to death was considered as overall survival (OS).

### Statistical analysis

The statistics of the study were made with the SPSS 25 program. Continuous variables were shown as median values (minimum and maximum), and categorical variables were shown as numbers and percentages. Binary logistic regression analysis was applied for factors predicting radiological complete response. Model fit was evaluated using the Hosmer and Lemeshow test. Kaplan Meier method was performed for survival analysis and curves.

### Conference presentation

This study was presented as a poster at 2020 San Antonio Breast Cancer Symposium.

### Statement of ethics

The local ethics committee approved this study at the Istanbul University Faculty of Medicine (Number: 229693). The study was designed retrospectively. Written informed consent from participants was not required in accordance with local/national guidelines.

## Results

### Patient characteristics and survival outcomes

**Figure 1:**
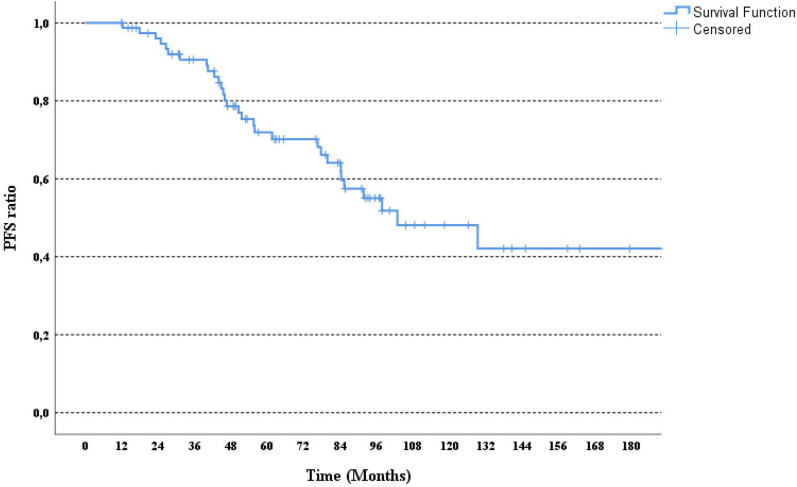
Kaplan Meier curve for PFS in patients who received long-term trastuzumab.

**Figure 2:**
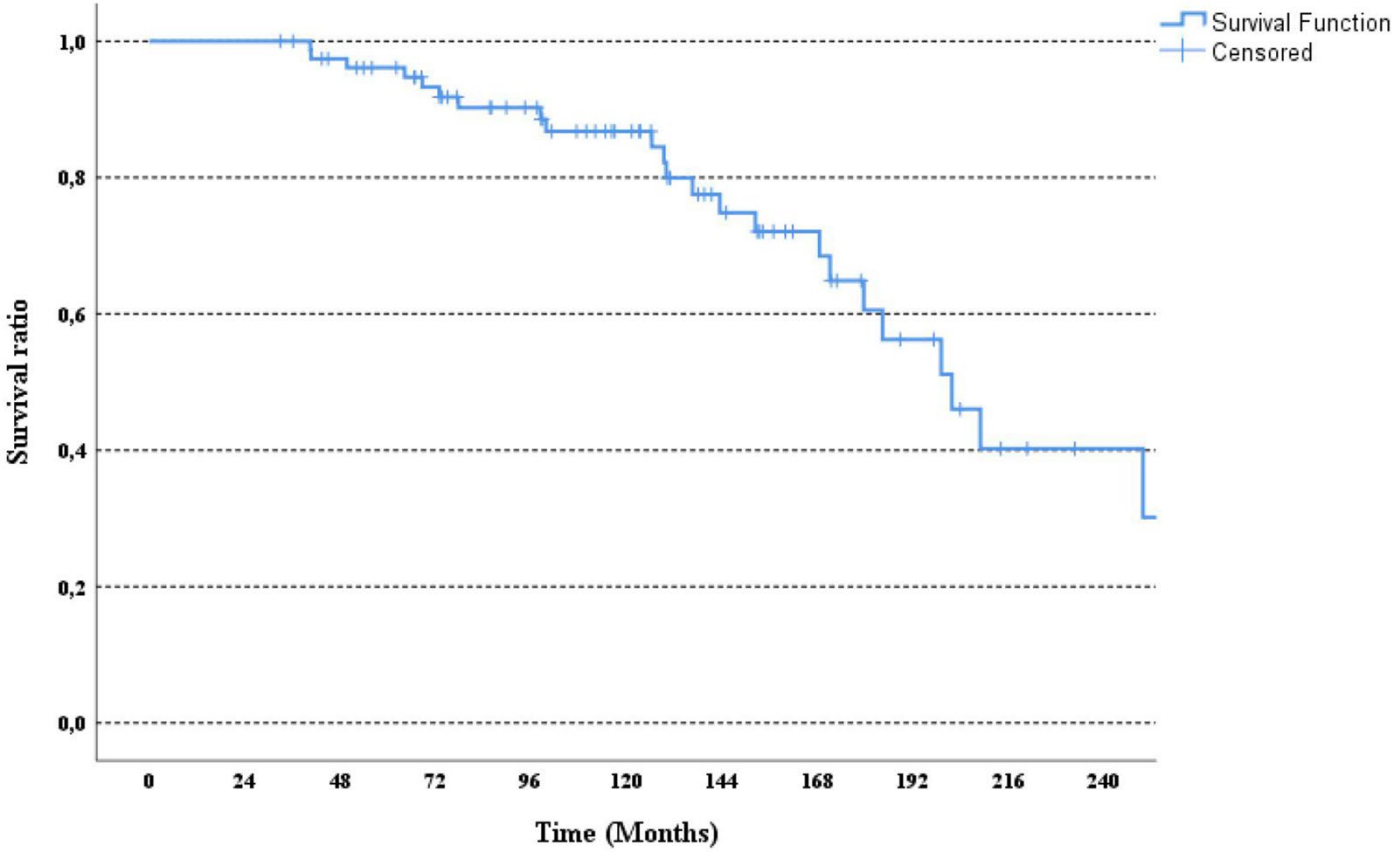
Kaplan Meier curve for OS in patients who received long-term trastuzumab.

Ninety-six patients who met the study inclusion criteria were identified, and 16 patients were excluded because of insufficient data. Analysis was performed with the data of eighty patients. The median age was 44 (22–68). Fifty-three patients (66.3%) presented with de-novo metastatic disease. The most common histopathological subtype was invasive ductal carcinoma (78.7%). ER positivity was present in 47 (58.8%) patients. HER2 positivity was mostly detected by the IHC (57%) method. The most common metastatic sites were bones (53.8%). Table 1 provides pathological and clinical summaries of the patients. The median follow-up period was 123 months. Trastuzumab treatment-related median PFS was found as 102 months (95% CI, 60–145) (Fig. 1). Five-, ten-, and fifteen-year survival rates were detected as 96%, 86%, and 60%, respectively (Fig. 2).

**Table 1:** Clinical and pathological characteristics of the patients.

	Number of patients	(%)
Age at diagnosis, years		
< 45	41	51.2
≥ 45	39	48.8
Menopausal status		
Premenopausal	53	66.3
Postmenopausal	27	33.7
Number of metastatic sites		
1	30	37.5
2	33	41.3
≥ 3	17	21.2
Metastatic sites		
Bone	43	53.8
Liver	22	27.5
Lung	21	26.2
Brain	12	15
Other sites	6	7.5
Stage at diagnosis		
Stage 1	7	8.7
Stage 2	23	33.7
Stage 3	23	33.7
Stage 4	27	33.9
Primary tumor location		
Left-sided	47	58.7
Right-sided	31	38.7
Bilateral	2	2.6
Histological type		
Invasive ductal carcinoma (IDC)	59	73.8
Other types	16	20
Unclassified	5	6.2
ER status at diagnosis		
Positive	47	58.8
Negative	33	41.2
PR status at diagnosis		
Positive	34	42.5
Negative	46	57.5
HER2 analysis method		
IHK Score 3	57	71.3
FISH positivity	23	28.7

### Treatment approach and analysis for complete response

Mastectomy was performed in 22 (27.5%) patients with disease control after de-novo metastatic disease. The median number of chemotherapy drugs used together with trastuzumab was 2 (1–6). In some patients, ER positivity was detected with recurrent biopsy, and 55 (68.8%) patients used at least one endocrine therapy agent together with trastuzumab. Palliative radiotherapy was applied to 43 (53.8%) patients, while metastasectomy was performed in 5 (6.3%) patients (Table 2). The median duration of trastuzumab use was 62 months (12–191). Complete response was detected in 60 (75%) patients. In the multivariate analysis to evaluate the factors predicting complete response; Menopause status (p = 0.03), number of metastatic sites (p = 0.01), and use of endocrine therapy with trastuzumab (p = 0.04) were detected to be statistically significant factors (Table 3). Cardiac toxicity occurred in only four (5%) patients receiving long-term trastuzumab-based therapy, and two (2.5%) had to discontinue trastuzumab.

**Table 2:** Treatment approaches for the patients.

	Number of patients	(%)
Mastectomy		
Before metastatic disease	53	66.2
After metastatic disease	22	27.5
No surgery	5	6.3
Number of chemotherapy drugs used with trastuzumab		
1	35	43.8
2	23	28.7
≥ 3	22	27.5
Endocrine therapy with trastuzumab		
Yes	55	68.8
No	25	31.2
Palliative radiotherapy with trastuzumab		
Yes	43	53.8
No	37	46.2
Metastasectomy		
Yes	5	6.3
No	75	93.7

**Table 3:** Logistic regression analysis for complete response in the HER2 positive breast cancer patients who were treated with long-term trastuzumab.

	Univariate analysis	Multivariate analysis	
	P-value	P-value	Odds ratio CI 95%
Age (≥ 45 vs. < 45)	0.2	0.8	
Menopausal status (post- vs. premenopausal)	**0.02**	**0.03**	**5.6 (1.1–28.1)**
De-novo metastasis (yes vs. no)	0.8	0.3	
HER2 positivity method (IHC Score 3 + vs. FISH)	0.4	0.1	
Number of metastatic sites	**0.003**		
≥ 3		**0.01**	**1**
1		**0.006**	**11.6 (2–67.3)**
2		**0.03**	**4.6 (1.1–19.8)**
Number of chemotherapy drugs with trastuzumab (1–2 vs. ≥ 3)	0.5	0.1	
Hormonotherapie with trastuzumab (no. vs. yes)	0.2	**0.04**	**6.4 (1.0–38.1)**
Palliative radiotherapy with trastuzumab (yes vs. no)	0.5	0.9	
Bisphosphonate with trastuzumab (yes vs. no)	0.7	0.9	

Significant values are in bold.

### Termination of trastuzumab

Trastuzumab-based treatment was discontinued in 15 (18.7%) patients after a durable complete response. Patients with hormone-positive diseases continued endocrine therapy after discontinuation of treatment, while patients with the hormone-negative disease were followed up without medication. Considering the general characteristics of the patients; The time to a radiological complete response with trastuzumab-based therapy was detected as 14.8 (2–55) months, and the median duration of trastuzumab use was 91 (20–191) months. The median follow-up period after discontinuation of trastuzumab was 32 months (11–66), and recurrence occurred in two (13.3%) patients whose treatment was discontinued during this period. Table 4 shows features of the patients whose trastuzumab-based therapy terminated. Overall, trastuzumab-based therapy was discontinued in patients with low tumor burden and generally had no visceral metastases at baseline.

**Table 4:** Characteristics of the patients whose trastuzumab treatment terminated.

	Number of patients	(%)
Age at diagnosis, years		
< 45	8	53.3
≥ 45	7	46.7
Menopausal status		
Premenopausal	10	66.7
Postmenopausal	5	33.3
Number of metastatic sites		
1	11	73.3
2	4	26.7
De-novo metastatic disease		
Yes	5	33.3
No	10	66.7
Metastatic sites		
Bone	9	60
Lymph node	4	26.7
Liver	2	13.3
Brain	1	6.7
Histological type		
Invasive ductal carcinoma	10	66.7
Other types	5	33.3
ER status at diagnosis		
Positive	6	40
Negative	9	60
PR status at diagnosis		
Positive	3	20
Negative	12	80
HER2 analysis method		
IHK Score 3	12	80
FISH positivity	3	20
Mastectomy		
Before metastatic disease	10	66.7
After metastatic disease	5	33.3
Endocrine therapy after trastuzumab termination		
Yes	6	40
No	9	60
Recurrence		
Yes	2	13.3
No	13	86.7

## Discussion

In the study, we showed that trastuzumab is effective and safe in patients who have used long-term trastuzumab-based therapy. In a pooled analysis of 2618 patients, the use of trastuzumab beyond progression was showed to be effective in patients who progressed in the first series with trastuzumab-based therapy^14^. In a study published by Daniels et al. was determined that approximately 26% of patients with metastatic HER2-positive breast cancer who were treated with trastuzumab lived longer than 5 years. In this study, the median age of the patients who lived long-term with HER2 targeted therapy was 54, the median HER2 targeted therapy duration was 58 months, and it was determined that the patients often had treatment breaks during the treatment process^15^. In multivariate analysis, we observed that radiological complete response was more common in patients who were premenopausal, had few metastatic organ involvements, and received endocrine therapy with trastuzumab. In a study published by Witzel et al., it was shown that the duration of progression after a complete response was longer in patients younger than 50 years of age who achieved long-term tumor remission under trastuzumab therapy, and in patients with good performance status^16^. In a study using data from patients in the CLEOPATRA study published by Hopkins et al. was determined that the number of metastatic sites and lactate dehydrogenase level before treatment were prognostic for OS and PFS in patients receiving trastuzumab, pertuzumab, and docetaxel^17^. In another study published by Steenbruggen et al., radiological complete response was found in 10% of the patients with HER2 positive metastatic breast cancer who were treated with trastuzumab-based therapy^18^. Also, it was showed that radiological complete response was found to be statistically significantly higher in patients with oligometastatic and single organ bone metastases.

In patients who have received long-term trastuzumab-based therapy with a durable a complete response that continues for a long time, discontinuation of trastuzumab and follow-up of the patient may be considered. In our study, these patients generally consist of patients with a low metastasis burden and no visceral metastases. In our study, the rate of relapsed patients after a median 3-year follow-up was found to be around 13%. In a multicenter study published by Niikura et al., the patients who received trastuzumab-based therapy for at least 2 years were evaluated; the complete response was detected in 52% of the patients with a median PFS of 11.2 years and a 5-year OS of 80%^19^. Trastuzumab treatment was terminated in 27 patients who received a median of 5.1 years of trastuzumab-based therapy, and progression was observed in 4 (14.8%) of the patients with a median follow-up of 3.9 years. In the case series of four diseases published by Takuwa et al., after clinical complete response to trastuzumab-based therapy, patients continued trastuzumab for 1–5 years as maintenance, and recurrence did not occur in the patients after 8–128 months of follow-up^20^. Also, in a letter to the editor published by Butterbaugh et al., cases published in the literature were pooled, and data from eight patients who achieved a durable complete response to trastuzumab-based therapy were shared; it was determined that complete response could continue for more than 12 years after the discontinuation of trastuzumab treatment^21^.

Due to the retrospective nature of our study, the patient group was heterogeneous. This may lead to patient selection bias in retrospective studies. The number of patients was limited because patients receiving long-term trastuzumab-based therapy were included in the study, and some data were missing in some patients.

## Conclusions

In the study, we showed the real-life outcomes of patients using trastuzumab-based therapy beyond progression. With trastuzumab-based therapy; We detected that premenopausal status, low disease burden, and using endocrine therapy together with trastuzumab predict complete response in HER2-positive metastatic breast cancer patients. It is unknown how long the treatment will be continued in patients who have a complete response to trastuzumab-based therapy and whose complete response continues for a long time. In this study, we show the results in a selected group of patients whose trastuzumab-based therapy was terminated. There are no defined criteria for termination of trastuzumab treatment in this selected patient group. Our study is one of the rare studies that contribute to the literature on this subject. It is necessary to confirm our data with multicenter studies involving a large number of patients. In the future, it is necessary to define well-researched criteria for the termination of cancer treatments after full recovery in metastatic cancer patients.

## Data Availability

This published paper contains all of the data produced or analyzed during this investigation.
